# Differential network interactions between psychosocial factors, mental health, and health-related quality of life in women and men

**DOI:** 10.1038/s41598-023-38525-8

**Published:** 2023-07-19

**Authors:** Martin Weiß, Marthe Gründahl, Jürgen Deckert, Felizitas A. Eichner, Mirjam Kohls, Stefan Störk, Peter U. Heuschmann, Grit Hein, the STAAB-COVID Study Group, Götz Gelbrich, Benedikt Weißbrich, Lars Dölken, Oliver Kurzai, Georg Ertl, Maria Barth, Caroline Morbach

**Affiliations:** 1 Translational Social Neuroscience Unit, Department of Psychiatry, Psychosomatic and Psychotherapy, Center of Mental Health, University Hospital Würzburg, Margarete-Höppel-Platz 1, 97080 Würzburg, Germany; 2 Institute of Clinical Epidemiology and Biometry, University of Würzburg, Würzburg, Germany; 3 Comprehensive Heart Failure Center, University Hospital Würzburg, Würzburg, Germany; 4 Department of Internal Medicine I, University Hospital Würzburg, Würzburg, Germany; 5 Clinical Trial Center, University Hospital Würzburg, Würzburg, Germany; 6 Institute of Medical Data Science, University Hospital Würzburg, Würzburg, Germany; 7 Institute for Virology and Immunobiology, University of Würzburg, Würzburg, Germany; 8 Institute for Hygiene and Microbiology, University of Würzburg, Würzburg, Germany; 9 University Hospital Würzburg, Würzburg, Germany

**Keywords:** Human behaviour, Quality of life, Anxiety, Depression

## Abstract

Psychosocial factors affect mental health and health-related quality of life (HRQL) in a complex manner, yet gender differences in these interactions remain poorly understood. We investigated whether psychosocial factors such as social support and personal and work-related concerns impact mental health and HRQL differentially in women and men during the first year of the COVID-19 pandemic. Between June and October 2020, the first part of a COVID-19-specific program was conducted within the “Characteristics and Course of Heart Failure Stages A-B and Determinants of Progression (STAAB)” cohort study, a representative age- and gender-stratified sample of the general population of Würzburg, Germany. Using psychometric networks, we first established the complex relations between personal social support, personal and work-related concerns, and their interactions with anxiety, depression, and HRQL. Second, we tested for gender differences by comparing expected influence, edge weight differences, and stability of the networks. The network comparison revealed a significant difference in the overall network structure. The male (*N* = 1370) but not the female network (*N* = 1520) showed a positive link between work-related concern and anxiety. In both networks, anxiety was the most central variable. These findings provide further evidence that the complex interplay of psychosocial factors with mental health and HRQL decisively depends on gender. Our results are relevant for the development of gender-specific interventions to increase resilience in times of pandemic crisis.

## Introduction

It is well known that physical and mental health are affected by psychosocial factors^1–4^, both in general and in times of crisis, e.g., a pandemic^5–7^. Factors like social support play a protective role^1,8,9^. In contrast, concerns (e.g., about one’s family or work) are related to deprivations in mental health^10–12^.

Social support has been broadly defined as “support accessible to an individual through social ties to other individuals, groups, and the larger community”^13^, i.e., having people to rely on who provide care, value, and love^14^. Social support is associated with lower levels of depression and anxiety^1,8^, particularly during times of crisis like the COVID-19 pandemic^7^. Previous studies differentiated between personal support, i.e., provided by family members and friends^6,15,16^, and work-related support, i.e., provided by supervisors and co-workers^17,18^. Both forms of social support have been associated with preserving and improving mental health and health-related quality of life (HRQL)^19–21^.

In contrast, concerns are generally associated with higher levels of perceived psychological stress^22,23^ and deteriorations of mental health and HRQL^10,12,24^. In one recent US-based study^10^, concerns about the well-being of one’s children or the health of other relatives were associated with decreased individual well-being during the COVID-19 pandemic^25^. Work-related concerns like job insecurity and financial concerns were associated with decreased mental health^12^ and overall well-being in employees^11^. In students, financial concerns were associated with self-reported anxiousness, nervousness, and sleep problems^26^. These results imply that social support is generally positively related to well-being and HRQL^27,28^, whereas financial, work-related, and personal concerns have negative effects^26,29,30^.

Previous studies have investigated whether social support and concerns have differential effects on mental health in women and men. Prior to discussing these differences, it is necessary to introduce sex and gender as two different concepts that are often conflated. The importance of differentiating between sex and gender in health and clinical research has been underlined repeatedly^31,32^. Sex, a biological construct, refers to the purely biological differences between males and females enabling sexual reproduction, whereas gender, a social construct, refers to the set of behaviors, interests, social conventions and roles associated with male or female sex^33,34^. Given the concept’s strong link to the social context^31,32^, the present study focusses on gender differences in psychosocial factors and mental health. Previous works have shown important gender-related differences in psychosocial and clinical variables. For instance, some studies suggest that women depend more upon personal support^35–37^, whereas men may derive more benefit from work-related support^38,39^. Women showed lower symptoms of depression and anxiety than men when perceiving social support provided by family members or friends^40,41^. In men, work-related support from co-workers and supervisions showed protective effects against depressive and anxiety disorders^38,39,42^. Elsewhere, this association of work-related support to mental health was stronger in men than in women^43,44^. Regarding concerns, previous studies found stronger deteriorating effects of work-related concerns on men’s health^45^, e.g., during job insecurity and unemployment^46^. Addressing personal concerns, women were more prone to psychological distress when neglecting their family due to their work^37^. Similarly, there were more work-family conflicts in mothers than fathers^47^, accompanied by factors like higher stress and lower life satisfaction^48^.

Inconsistent with these findings, other studies revealed comparable effects of social support and concerns in women and men of different ages^2,27,28^. For example, a study investigating effects of social support on self-reported physical and mental health in Slovak adolescents (*N* = 2616, mean age = 15) found lower social support and higher (mental) health in boys compared to girls, yet no gender difference in the positive relation between social support and health^27^. Hann et al.^28^ found a gender-independent negative relation of social support and depressive symptoms in cancer patients. In a large cross-sectional data set of working European adults (*N* = 40,089), work-related support mediated the negative association between work-related stress and acute mental well-being and health similarly in women and men^2^. Work-related concerns like job insecurity, resulting financial concerns, and psychosocial stress at work also had negative effects on mental health in both men and women^9,11^.

In summary, it remains unclear whether psychosocial variables affect men’s and women’s mental health differently. Moreover, the different dimensions of social support and concerns (personal and work-related) were previously assessed in separate studies or analyses, without accounting for the complexity of their interrelations as well as their associations with mental health and HRQL.

To overcome these limitations, we applied network analyses to quantify the complex associations between important psychosocial variables (personal and work-related support; personal and work-related concerns), mental health (i.e., anxiety and depression), and HRQL in women and men during the COVID-19 pandemic. In comparison to traditional methods such as regression analysis and factor analysis, network analysis allows for the examination of complex relationships between multiple variables simultaneously, providing a more comprehensive understanding of the factors influencing mental health^49^. This is particularly important given the intricate nature of mental health. Mental health often involves numerous interconnected factors^50^. By analyzing the interrelations between different psychosocial variables, network analysis can contribute to the identification of the most influential factors affecting mental health^51,52^. Identifying these central factors can help prioritize interventions and allocate resources more effectively^53^.

Inspired by previous work^6,17,22^, we hypothesized that work-related and personal support as well as work-related and personal concerns are core components in a network of psychosocial and psychopathological variables directly connected with individual differences in anxiety, depression, and HRQL, especially during the COVID-19 pandemic. We predicted positive relations between social support and mental health, and negative relations between concerns and mental health^10,12,15^. These associations might be comparable between women and men^2,27,28^. Alternatively, inspired by studies showing gender differences^35,39,43^, work-related concerns and support might be more closely associated with mental health in men, while personal concerns and support should be more influential in women.

### Network analysis

As indicated above, we applied a network approach to test these assumptions^54,55^. The proponents of network theory in clinical psychology assume that mental disorders arise from complex relationships between symptoms and thus conceptualize psychiatric disorders as emergent phenomena^56^. For instance, when taking a network perspective on psychopathology, depression constitutes a dynamic network of interdependent symptoms^57^. Analyses based on a network approach allow a graphical representation of all variables as individual nodes. This helps identify variables particularly associated with others, such as links between symptoms of different disorders that could explain comorbidities^56,58^. Several studies have used this method to address psychiatric research questions, e.g., examining relations between neighborhood social environment and symptoms of paranoia^59^, the impact of the COVID-19 pandemic on mental health^60^, or associations between items measuring individual differences in depression and anxiety^61,62^. We therefore applied a network approach to investigate the hypothesis that during the first year of the COVID-19 pandemic, the complex interplay between concerns, social support, anxiety, depression, and HRQL is modified by gender^63–66^.

## Methods

### Sample

The Characteristics and Course of Heart Failure Stages A–B and Determinants of Progression (STAAB) study is a cohort of 5010 randomly sampled volunteers of the general population of Würzburg, Germany recruited between 2013 and 2017 (source population 124,297 inhabitants as of 2011 census). Inclusion of STAAB participants is restricted to 30–79 years of age at the baseline measurement and sampling was stratified 1:1 for gender and 10:27:27:27:10 for age groups of 30–39/40–49/50–59/60–69/70–79 years. In order to ascertain a continuously balanced recruitment, invitations to the study were sent out in batches that were iteratively re-adjusted for gender and pre-defined age groups according to the respective response rates. The complete study design and the rationale of the STAAB cohort is described in detail elsewhere^67^. Here, we analyze data from the STAAB-COVID-One program, which assessed the psychosocial impact of the COVID-19 pandemic of participants of the STAAB cohort during the first year of the pandemic. On-site participation including blood samples was performed in the joint study line of the German Center for Heart Failure (DZHI) and the Institute for Clinical Epidemiology and Biometry (IKE-B) in Würzburg. Afterwards, participants answered the survey items used in the present study at home via online survey or a paper-based questionnaire. Data were collected between June 17th and October 18th 2020^68^. Based on evidence from previous work as reported above, we inserted social variables capturing personal support and concern^6,17,22^ as well as work-related support and concern^35,39,43^ into the ongoing study (see “Measures” for more details). All procedures within the STAAB program are subject to a rigorous quality control, follow predefined standard operating procedures, and comply with the Declaration of Helsinki. The study protocol and procedures were approved by the ethics committee of the medical faculty of the University of Würzburg (vote #98/13) and data protection office (#J-117.605-09/13). The final sample size for the present analysis included 2890 participants (1520 female, 1370 male; note that there was no option to identify as non-binary) with a mean age of 59.6 (*SD* = 11.19, range 34–85). All participants provided written informed consent prior to any study examination.

### Measures

To test our research question, we applied for access to variables that (A) assess HRQL and important indicators of mental health and (B) variables that address personal and work-related concern as well as support. In the STAAB survey, mental health outcomes were measured using validated scales for depression (Patient Health Questionnaire-9; PHQ-9)^69^ and generalized anxiety (Generalized Anxiety Disorder Scale, GAD-7)^70^. For both the PHQ-9 and the GAD-7, a score ≥ 10 indicates clinically relevant levels of depression and anxiety, respectively. HRQL was assessed using the visual analogue scale of the EQ-5D (range 0–100)^71^. Higher scores indicate higher HRQL.

To document personal support, we used variables assessing perceived social support and emotions associated with family members (“How strongly do you feel supported by your social environment?”, “How do you feel towards the individuals in your family/ in the household at the moment?”, rated on a 5-point Likert scale ranging from 0 = “very bad” to 4 = “very good”; “Did your feelings towards your family member/ individuals in your household change since the COVID-19 pandemic?”, rated on a 3-point scale with 0 = “deteriorated”, 1 = “unchanged”, and 2 = “improved”). For work-related support, we used variables assessing perceived social support and emotions associated with co-workers and supervisors (“How strongly do you feel supported by your colleagues?”, “How strongly do you feel supported by your supervisor?”, “How do you feel towards the individuals at work at the moment?”, rated on a 5-point Likert scale from 0 = “very bad” to 4 = “very good”; “Did your feelings towards the individuals at work change since the COVID-19 pandemic?”, rated on a 3-point scale with 0 = “deteriorated”, 1 = “unchanged”, and 2 = “improved”). To capture personal concern, we used three items related to “burden of caring for children, parents, or other family members”, “having no one to discuss the problems with”, and “burdens due to contact bans with older people (e.g., parents, grandparents, etc.)”. For work-related concern, we used two items targeting “stress at work or school” and “financial problems or worries”. Concern variables were assessed using a three-point Likert-scale (0 = “not impaired”, 1 = “lowly impaired”, 2 = “heavily impaired”). The internal consistencies (Cronbach’s α) of PHQ, GAD, personal support, work-related support, personal concern, and work-related concern were 0.85, 0.87, 0.59, 0.78, 0.49, and 0.44, respectively.

### Statistical analyses

All statistical analyses were conducted using *R* Version 4.0.4. The structure of associations between the included variables (i.e., personal support, work-related support, personal concern, work-related concern, depression, anxiety, HRQL, age) was analyzed using a regularized partial correlation network. From this network, we identified the most central variables, their predictability, network stability, and inference accuracy^72,73^. To investigate gender differences, we conducted two separate network analyses for women and men, respectively. In addition, we calculated a joint network of women and men to compare the results with the differential networks.

#### Network estimation

We estimated the network structures via Mixed Graphical Models (MGMs) using the *R*‐packages *mgm*^74^ and *bootnet*^73,75^, as MGMs explicitly account for mixed measurement scales of variables. “Nodes” describe the individual variables (e.g., depression symptoms) while the “edges” describe the statistical relationships between them. The so-called “centrality index” determines the importance of each node within the network based on the strength of its connections to other nodes. Nodes and edges were computed with the Fruchterman-Reingold algorithm from the *R*-package *qgraph*^75^. To investigate how support and concern variables were associated with mental health symptoms (depression, anxiety) and HRQL, all seven variables were included into a Gaussian graphical model (GGM)^76^. Here, a connection between two nodes represents the connection after controlling for all other edges in the network, similar to partial correlations. For the seven variables (i.e., seven nodes), we estimated 21 pairwise parameters for each network. We employed a penalty approach to control for potential spurious associations which would lead to false-positive findings, namely the least absolute shrinkage and selection operator (LASSO)^77^. LASSO shrinks edge weights, reducing smaller edges to zero and usually resulting in a sparse structure. To choose the most appropriate network, we used the Extended Bayesian Information Criterion^78^, setting its hyperparameter to default (γ = 0.25). We tested for redundant nodes using the ‘goldbricker’ function from the *networktools R*-package^79^. No statistically redundant nodes were indicated. For visualization of edge weights, we consistently used the value of the highest edge weight across a network for the whole (= joint) sample, the female subset, and the male subset (*w* = 0.678) to ensure a homogenous scaling of edge depictions across all networks.

#### Network inference

We report two main parameters for assessing nodes^80,81^. First, we calculated the expected influence (EI) as centrality index for the female and male networks^82^. The EI captures the degree of connectivity of a specific node with the network’s other nodes. Its value represents the sum of all edge weight values that are interconnected with a particular node^83^. Second, predictability estimates were derived for all variables in the networks and implemented visually as pie charts around each node. Predictability quantifies the variance of a node explained by all its neighbors measured as R^2^^72^. We also conducted difference tests to compare individual centrality estimates as a non‐parametric bootstrapping method with 2000 iterations.

To visually compare female and male participants, we created a difference network based on the difference weights matrix. The matrix was calculated by subtracting the female from the male subsample weight matrix. To compare both networks, we performed the non-parametrical permutation-based Network Comparison Test (NCT)^84^ with 1000 permutations. As the method compares multiple edges between networks, we applied Bonferroni adjustment for multiple comparison correction.

#### Network accuracy and stability

For the female and male networks, edge weight stability was tested by bootstrapping 95% confidence intervals (CI) of the edge weights (Fig. S3). The order stability of the centrality measures was tested by subset bootstrap procedures, so that the central node should remain central after dropping random participants (Fig. S4)^85^. As index, we estimated the centrality stability (CS coefficient), which should be at least 0.25 and preferably above 0.5. Further accuracy and stability analyses for this network, i.e., edge weight significance tests (testing for significant differences for all edges) and centrality difference tests (testing for centrality differences for all nodes), can be found as Supplementary Figs. S4 and S5.

#### Confirmatory analysis

Finally, we tested the split‐half reliability as a confirmatory analysis of the gender-specific networks. Using the *psychometrics R*-package^86^, we estimated a GGM in a randomly-drawn half of the female and male dataset, respectively, and then fitted a confirmatory network model to the second half.

### Ethical standards

The authors assert that all procedures contributing to this work comply with the ethical standards of the relevant national and institutional committees on human experimentation and with the Helsinki Declaration of 1975, as revised in 2008.

## Results

### Sample characteristics

3034 persons participated in the STAAB-COVID-One program, providing blood sample and questionnaire data. Of those participants, 2892 provided complete questionnaire data, but two were excluded as they did not report their gender. Thus, 2890 (60%) of the 4860 participants remaining in the study provided complete questionnaire data (53% female; mean age women = 59, *SD* = 11.0; mean age men = 60, *SD* = 11.4). Descriptive statistics for the analyzed variables are presented in Table 1. The prevalence for depression (PHQ) and anxiety (GAD; cut-off values ≥ 10) were 8% and 4%, respectively.

**Table 1:** Descriptive statistics.

	Women (*N* = 1520)		Men (*N* = 1370)		*p-*value
	*N*	*M* (*SD*) or %	*N*	*M* (*SD*) or %	
Age	1520	59.10 (11.00)	1370	60.14 (11.37)	0.013
Depression	1464	4.27 (3.77)	1329	3.10 (3.31)	< 0.001
Prevalence		10		5	
Anxiety	1469	3.00 (3.32)	1327	2.15 (2.85)	< 0.001
Prevalence		6		3	
HRQL	1462	76.22 (17.00)	1324	76.27 (16.78)	0.930
Personal concern	1440	1.00 (1.23)	1313	0.67 (1.02)	< 0.001
Work-related concern	1453	0.54 (0.74)	1323	0.43 (0.66)	< 0.001
Personal support	1184	7.14 (1.61)	1089	7.31 (1.51)	0.008
Work-related support	759	8.50 (2.74)	625	8.78 (2.74)	0.054

HRQL = health-related quality of life; prevalence for depression and anxiety was calculated using a sum-score cutoff of ≥ 10 and is indicated in percent. The comparison between women and men was conducted using *t*-tests.

### Analyzing network nodes

**Figure 1:**
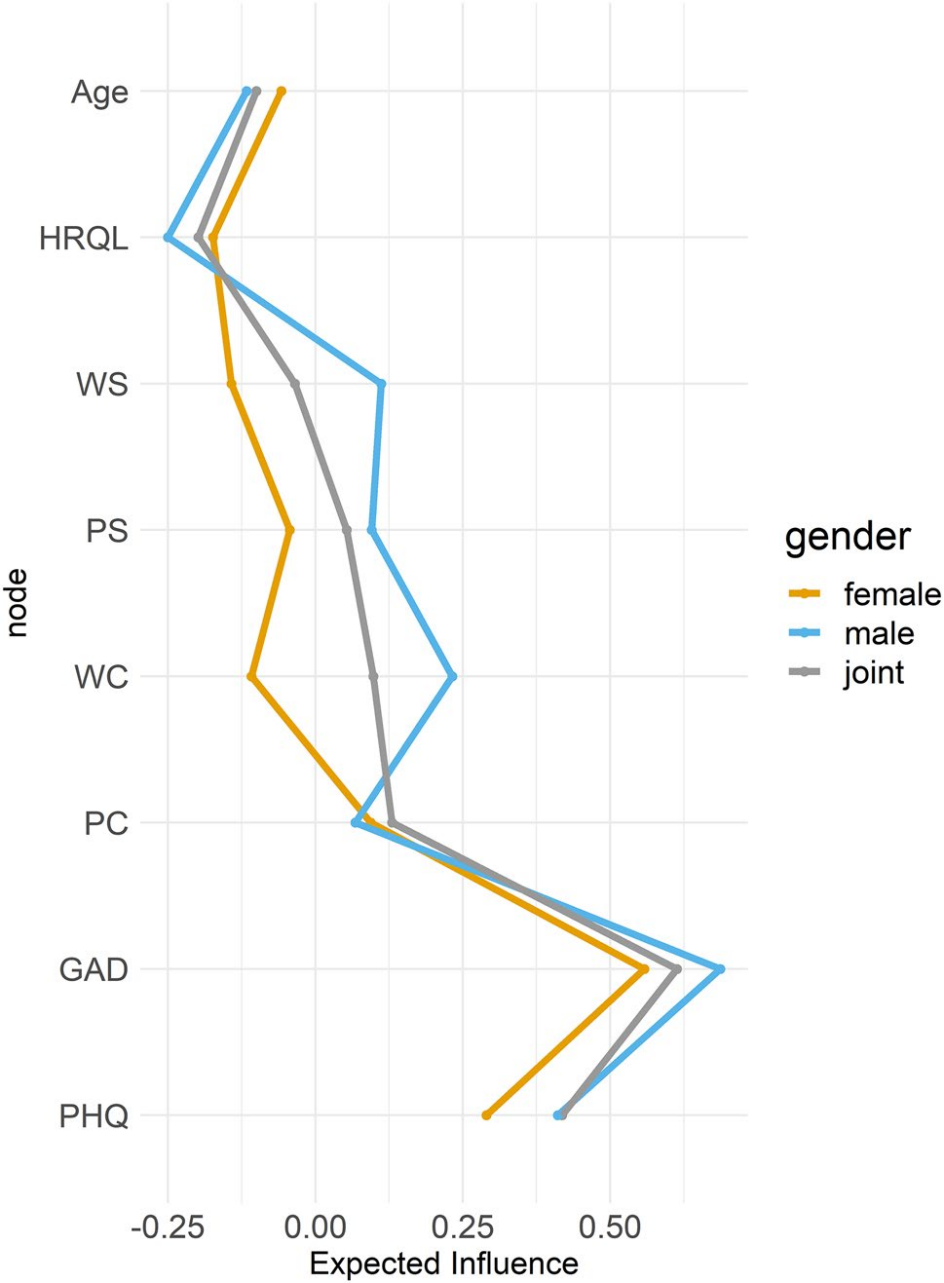
Standardized expected influence (EI) for different nodes in the joint, female, and male network. Larger EI values (represented on the x-axis) indicate higher centrality to the network/ higher predictability. The y-axis represents the name of the specific node. *HRQL* health-related quality of life, *GAD* anxiety, *PHQ* depression, *WS* work-related support, *PS* personal support, *WC* work-related concern, *PC* personal concern.

Analyzing centrality, i.e., the importance of a node for the connectivity of the network, GAD showed the highest EI (0.61) in the joint network, in the male (0.69), and in the female network (0.56; Fig. 1). In all networks, HRQL had the most negative EI (joint = − 0.20; women = − 0.17; men = − 0.25).

To secure these results, we tested the accuracy of the network and order stability of the centrality measures. According to the width of the CIs, the bootstrapped edge weight analysis suggests that the models were estimated accurately (Fig. S2). The CS‐coefficients of 0.75 suggest a stable order of centrality^85^. The centrality difference tests indicate that EI significantly differs between most nodes (Fig. S4). For men and women, the node with the largest EI, GAD, was significantly larger than all other nodes except PHQ. Additional edge weight significance tests are illustrated in Fig. S5.

Average node predictabilities in the female and male network were 0.36 and 0.35, respectively. The nodes with the highest predictability in both networks were PHQ (female R^2^ = 0.69, male R^2^ = 0.67) and GAD (female R^2^ = 0.67, male R^2^ = 0.66). The node with the lowest predictability were age (female R^2^ = 0.02, male R^2^ = 0.04) and HRQL (female R^2^ = 0.31, male R^2^ = 0.23).

### Analyzing network structures

**Figure 2:**
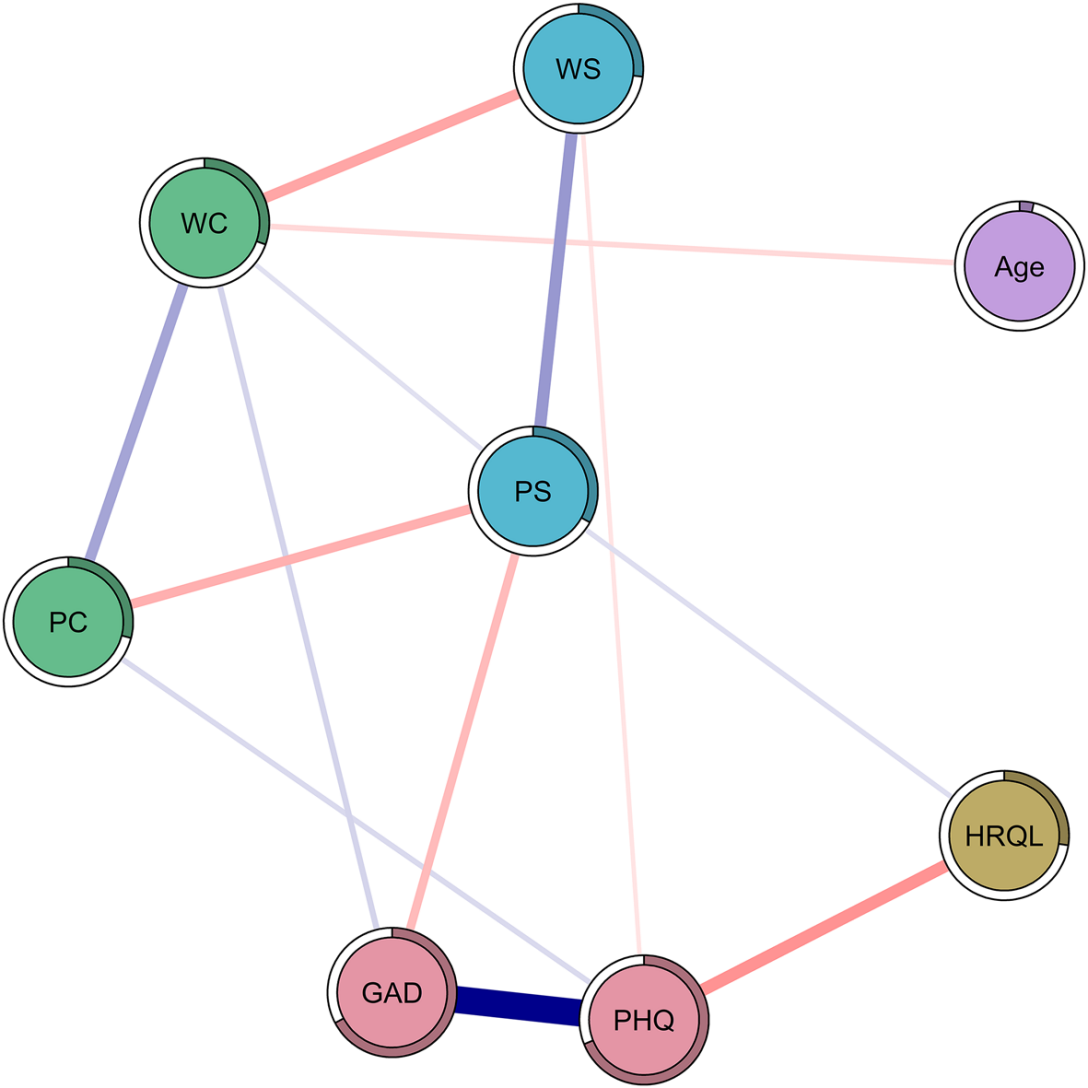
Joint network of all participants in the present study. Nodes represent core features; edges represent the regularized partial correlation between any two nodes. Positive edges are indicated by blue lines, negative edges are printed in red. Thicker and more saturated edges visualize stronger connections. Stronger and/or more connections are placed closer to one another. *HRQL* health-related quality of life, *GAD* anxiety, *PHQ* depression, *WS* work-related support, *PS* personal support, *WC* work-related concern, *PC* personal concern.

Figure 2 illustrates the network structure of the joint network. The strongest positive associations were found between GAD and PHQ (*w* = 0.68), work-related and personal support (0.27), and work-related and personal concern (*w* = 0.24). The strongest negative associations were found between depression and HRQL (*w* = − 0.29), work-related concern and work-related support (*w* = − 0.24), personal concern and personal support (*w* = − 0.21), and GAD and personal support (*w* = − 0.18). The average node predictability in the joint network was 0.36, i.e., a moderate level of predictability compared with other psychopathology networks^72^. The nodes with the highest predictability were PHQ (R^2^ = 0.69) and GAD (R^2^ = 0.67). The node with the lowest predictability was age (R^2^ = 0.04).

A formal network comparison revealed a significant gender effect in the omnibus test on network structure (max. difference in edge weights = 0.255, *p* = 0.004, 1000 permutations), with comparable overall connectivity strength (global strength difference = 0.04; male sample network strength = 2.34; female sample network strength = 2.38, *p* = 0.891, 1000 permutations). Work-related concern and support had higher EI scores in the male network (concern: *p*_uncorrected_ = 0.032; *p*_corrected_ =  > 0.99; support: *p*_uncorrected_ = 0.045; *p*_corrected_ = 0.464; Fig. 1).

**Figure 3:**
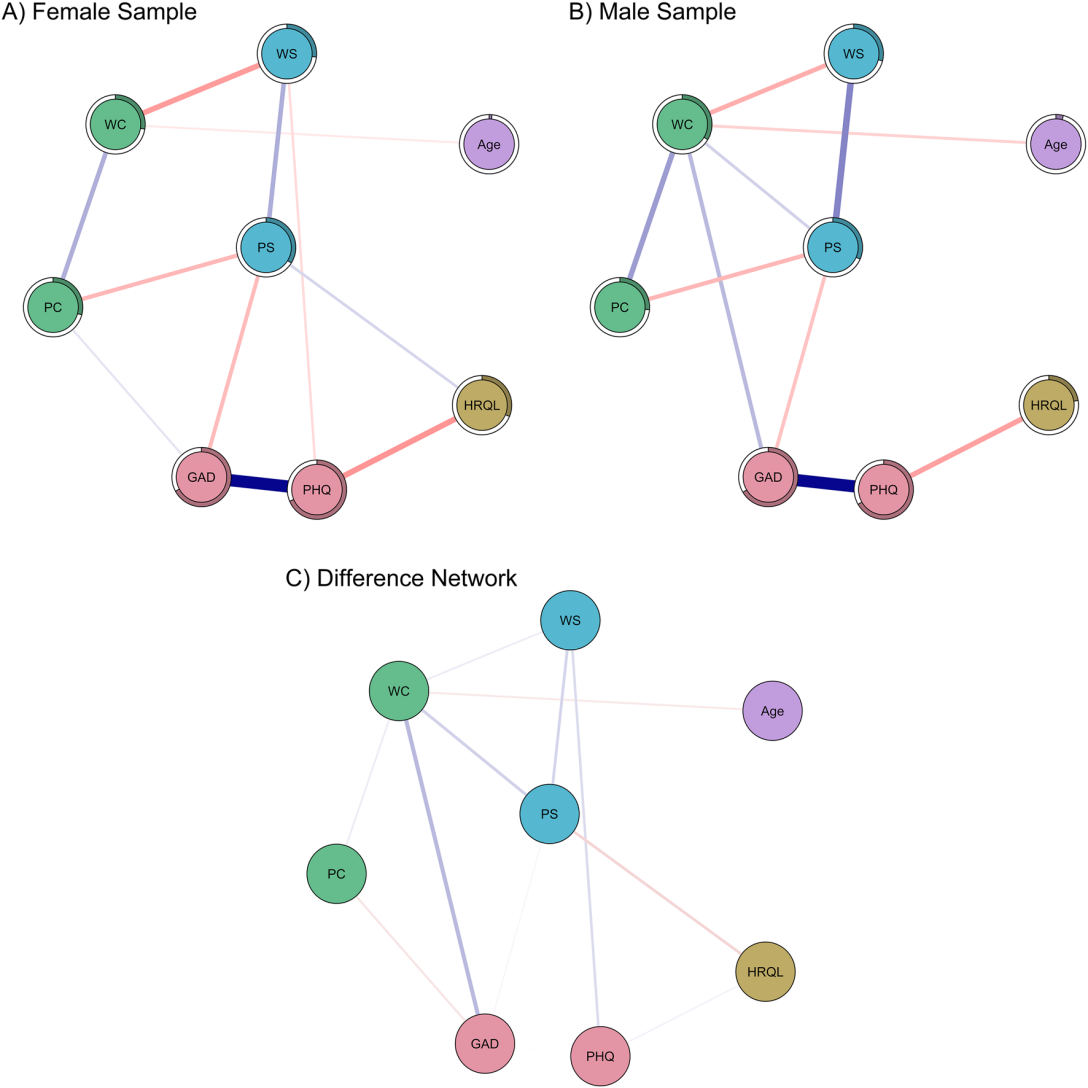
Psychological (mental) health network for the female (**A**; *n* = 1520) and male (**B**; *n* = 1370) sample. Nodes represent core features, edges represent the regularized partial correlation between any two nodes. Positive edges are indicated by blue lines, negative edges are printed in red. Thicker and more saturated edges visualize stronger connections. Stronger and/or more connections are placed closer to one another. Panel C depicts the difference between male and female networks. Blue lines indicate a positive value for the difference between men and women, whereas red lines indicate a negative value for the difference. *HRQL* health-related quality of life, *GAD* anxiety, *PHQ* depression, *WS* work-related support, *PS* personal support, *WC* work-related concern, *PC* personal concern.

The network structure of the female and male subsamples and the difference network are illustrated in Fig. 3. They reveal significant gender differences regarding both interconnectivity and relations with mental health outcomes. A gender effect in the connectivity between GAD and work-related concern (Bonferroni corrected *p* = 0.028; Fig. 3c) reflected a positive association between GAD and work-related concern for men (*w*_male_ = 0.19; Figs. 3b and S1b), but not for women (*w*_female_ = 0, Figs. 3a and S1a). Descriptively, the negative association between work-related concern and personal support (*w*_female_ = 0, *w*_male_ = 0.12, *p*_uncorrected_ = 0.143, *p*_corrected_ > 0.99) was only present in the male, but not in the female network. The negative association between work-related support and PHQ (*w*_female_ = -0.09, *w*_male_ = 0, *p*_uncorrected_ = 0.226, *p*_corrected_ > 0.99) as well as the positive associations between personal concern and GAD (*w*_female_ = 0.07, *w*_male_ = 0, *p*_uncorrected_ = 0.290, *p*_corrected_ > 0.99) and personal support and HRQL (*w*_female_ = 0.11, *w*_male_ = 0, *p*_uncorrected_ = 0.193, *p*_corrected_ > 0.99) were only present in the female, but not the male network.

Apart from these differences, both women and men showed strong positive associations between work-related and personal concern (*w*_female_ = 0.21, *w*_male_ = 0.26), GAD and PHQ (*w*_female_ = 0.67 and *w*_male_ = 0.66), and work-related and personal support (*w*_female_ = 0.22, *w*_male_ = 0.33). The strongest negative associations were found between PHQ and HRQL (*w*_female_ = − 0.28, *w*_male_ = − 0.25) and personal concern and personal support (*w*_female_ = − 0.19, *w*_male_ = − 0.19).

The split-half reliability analysis suggested a fit with a root mean square error of approximation of 0.05 (95% CI 0–0.097) for women, a fit of 0.08 (95% CI 0.04–0.12) for men, and a comparative fit index of ≥ 0.98 for both women and men^87^.

## Discussion

Psychosocial factors like social support and financial, work-related, or personal concerns are related to positive and negative states of mental health and quality of life^6,20,88^. However, data investigating whether these relationships are the same for men and women are lacking. We therefore used network analysis and a large dataset from the general population of Würzburg collected during the first year of the COVID-19 pandemic to investigate the complex associations between social support, concerns, mental health, and health-related quality of life in women and men.

In general, we hypothesized that work-related and personal support as well as work-related and personal concerns would be central nodes in our psychosocial and psychopathological network. We expected positive associations between social support and mental health, and negative associations between concerns and mental health^6,17,22^. Extending previous research, we further inspected the dependence of these effects and interrelations on gender. Some previous research suggests comparable associations between women and men^10,12,15^, whereas other findings suggest stronger associations of mental health with work-related concerns and support in men and with personal concerns and support in women^35,39,43^.

Independent of gender, anxiety was the most central network variable. However, the analysis of structural network connectivity revealed that work-related concerns connect differently with mental health outcomes in women and men. In more detail, increasing anxiety scores were associated with increasing work-related concerns in men, while there was no effect of work-related concerns in women. Instead, increasing anxiety scores were associated with increasing concerns regarding family and friends. Moreover, women showed a positive relation between personal support and HRQL, indicating an increase in quality of life if perceived social support provided by friends and family is high.

The gender-specific effects of work-related concern agree with previous results showing that men perceive higher work-related psychological distress^9^ and are more affected by job insecurity and unemployment^46^, while women report higher distress when neglecting their family due to their work^37,47,48^. The finding of stronger work-related associations in men and stronger family and friend-related associations in women may be traced back to traditional gender norms and roles. Traditionally, the stereotypical male gender role focuses on work and providing resources for the family and the female gender role focuses on caregiving^89,90^. A review on gender and mental health^90^ proposes that stressors are more harmful in those domains that are more closely associated with one’s gender role, i.e., work for men and family for women, in line with our findings. In contrast to our findings, some previous research found no gender differences in the effect of work-related concerns on mental health^9,11,45^, but these studies used a less detailed statistical approach compared to our network analyses. In recent research during the COVID-19 pandemic, female employees perceived the financial consequences of the pandemic as less extreme than male employees^91^, further supporting a higher relevance of work-related concerns for men’s mental health^46^. There was no significant gender difference in the negative association between personal concerns and psychopathology^10,92^. Moreover, although protective effects of social support against mental health deprivations are well known, especially during the pandemic^6,17,18^, work-related and personal support did not play a central role in our networks. Note also that both men and women showed a negative relation between personal support and mental health, i.e., anxiety. Previous findings have implied a stronger dependency of women compared to men on social support for mental health enhancement^35,40^. In our study, a gender-specific effect emerged for personal support and HRQL: personal support by family and friends was associated with higher HRQL in women, while this relation was absent in men. This once again aligns with the traditional female family-related role, which includes a higher tendency to engage in close social contacts and seek social support for increased well-being and stress reduction^93,94^. Moreover, this stronger role of social support in women even emerged beyond the family and friend context, as women showed lower depression with higher work-related support. Thus, with regard to the work context, men’s mental health was negatively associated with work-related concerns, whereas women’s mental health may profit from work-related support.

For both men and women, connectivity analyses showed strong positive association between work-related and personal concerns as well as work-related and personal support. In line with previous studies independent of a pandemic context^95–97^, we found a positive relationship between trait anxiety (GAD) and depression (PHQ), and a negative relationship between depression and HRQL during the COVID-19 pandemic. Moreover, the joint network revealed that personal support was associated with decreasing anxiety, further underlining the mental health-increasing effect of personal support^1,7,8^.

There are some limitations to consider when interpreting the results. We conducted a cross‐sectional analysis of a representative age and gender-stratified sample aged 34 to 85 years at baseline investigation drawn from the city of Würzburg located in Southern Germany. Therefore, the findings may not be transferable to populations with a different age context, location, or ethnic background. Also considering that previous research indicated differences in network connectivity in different populations^98^, the findings may not be generalizable to clinical samples. In addition, the 8% prevalence of depression and 4% prevalence of anxiety are in line with other population-based surveys in Germany^99–101^, but disagree with recent research indicating increases in depression and anxiety scores to 11% during the pandemic^102^. Note also that our data was obtained during the COVID-19 pandemic, i.e., a context of increased impairments in mental health^103,104^. Given that the COVID-19 pandemic provided a very specific context, it still needs to be clarified if our results generalize to other pandemic-independent situations. Future studies should validate our findings in more heterogeneous samples, clinical samples, and a non-pandemic context. Moreover, the cross-sectional design does not allow us to test whether the observed associations are affected by the pandemic. Finally, to test our a priori hypotheses, we only analyzed a selection of potentially relevant variables. Statistical models are solely able to inform about variance between items included in the model. As the COVID-19 pandemic resulted in very individual concerns and support strategies^105^, it was not trivial to decide which variables should be integrated into such a complex system. Therefore, confirmatory research is needed to validate the importance of other factors in psychopathological networks. Finally, future studies should collect socio-economic data, as this would enable a comprehensive understanding of the complex interplay between socio-economic factors, psychosocial variables and psychological well-being, facilitating the development of targeted interventions and policies.

Overall, our study supports the need to consider psychosocial factors and psychopathology as an interrelated network of protective and detrimental influences. As one of the first studies, we point out gender-related differences within these networks while also highlighting the influence of psychosocial factors. Our findings underline the importance of targeting social aspects in therapeutic interventions for the improvement of women’s and men’s mental health.

## Supplementary Information

Supplementary Figures.

## Data Availability

The data that support the findings of this study are available on request from the corresponding author. The data are not publicly available due to data protection restrictions.
