# Supplementary material for: Differential network interactions between psychosocial factors, mental health, and health-related quality of life in women and men
Source: Sci Rep. 2023 Jul 19;13:11642. doi: 10.1038/s41598-023-38525-8 (PMC10356800; doi:10.1038/s41598-023-38525-8)
Supplement: Supplementary file 1 — Supplementary Figures. [file 41598_2023_38525_MOESM1_ESM.pdf]

Supplementary Material

Differential network interactions between psychosocial factors, mental health, and health-related quality of life in women and men

Martin Weiß, Marthe Gründahl, Jürgen Deckert, Felizitas A. Eichner, Mirjam Kohls, Stefan Störk,

Peter U. Heuschmann, & Grit Hein on behalf of the STAAB-COVID study group

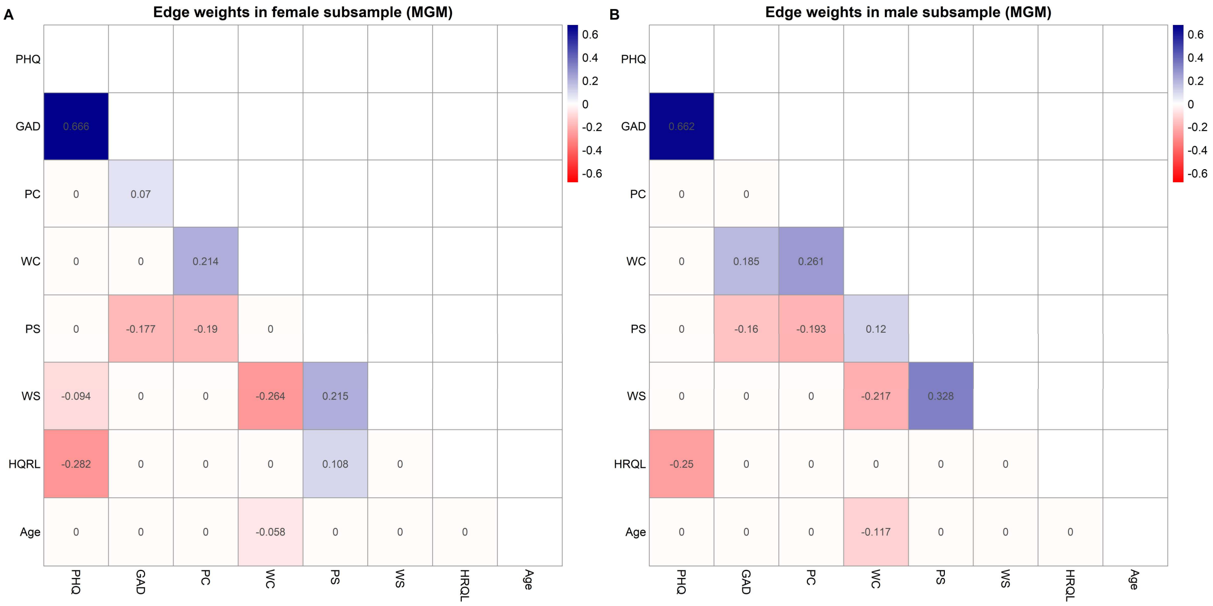

Figure S1. Weight matrices for women (left) and men (right) derived from computational network modelling with a mixed graphical model. HRQL = health-related quality of life, GAD = anxiety, PHQ = depression, WS = work-related support, PS = personal support, WC = work-related concern, PC = personal concern.

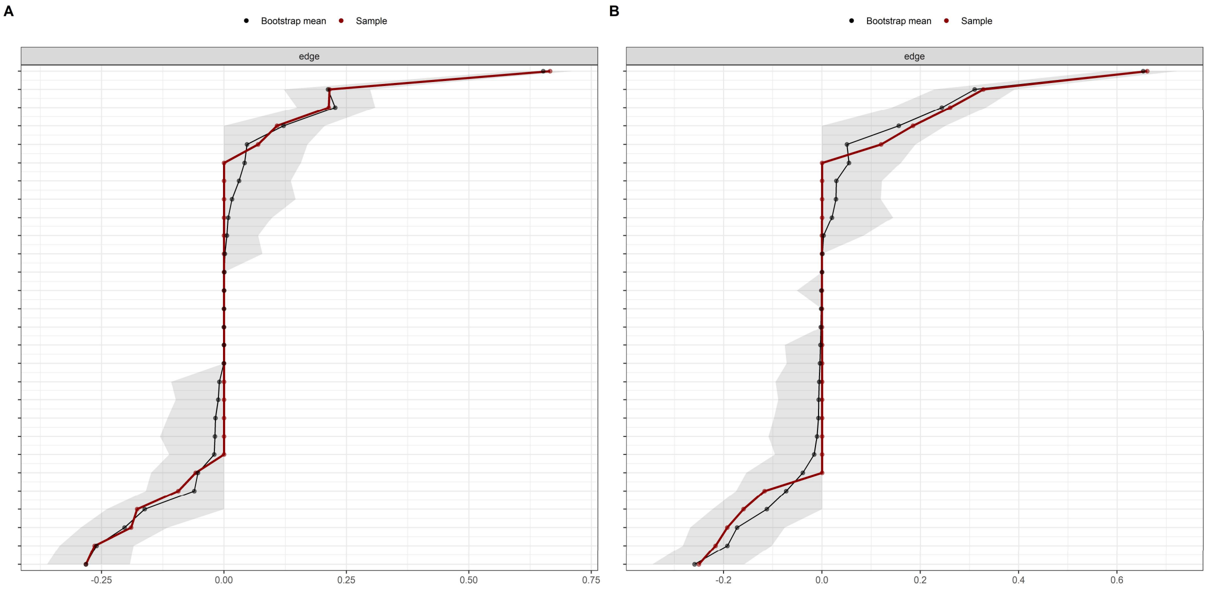

Figure S2. Bootstrapped confidence intervals (CIs) of the edge weights in the female (left) and male (right) network. The red line indicates the edge weight values and the gray area the bootstrapped 95% CIs. Each horizontal line represents one edge of the network, ranging from the edge with the highest edge-weight (top) to the edge with the lowest edge-weight (bottom). In case of tie (for instance, multiple edge-weights were estimated to be exactly 0), the mean of the bootstrap samples was used in ordering the edges.

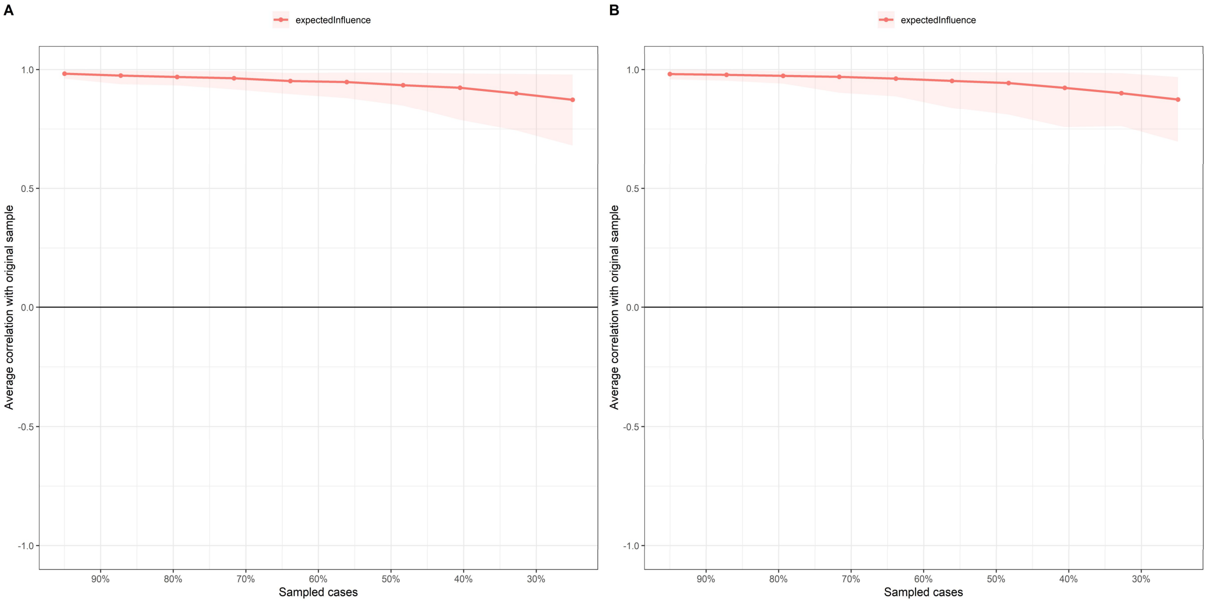

Figure S3. Subset bootstrap procedure for the psychological health networks for women (left) and men (right) showing the average correlations between centrality indices of the original network constructed on the full data with networks estimated on samples with fewer participants.

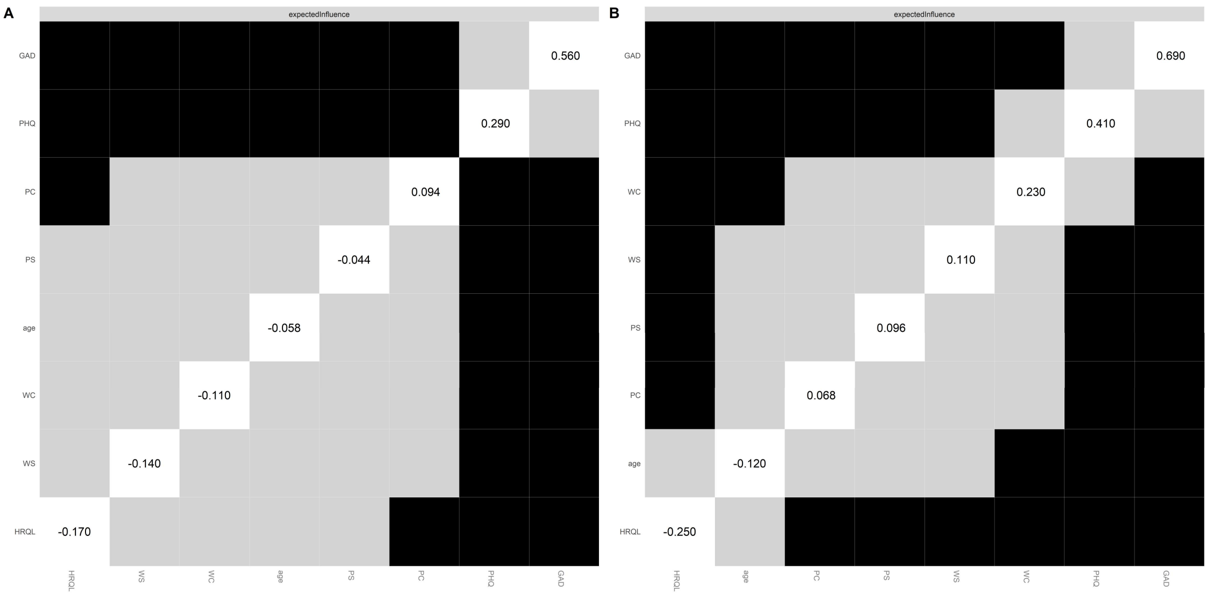

Figure S4. Degree centrality difference test for the female (left) and male (right) network. Gray boxes indicate nodes or edges that do not differ significantly from each other, black boxes represent nodes or edges that do differ significantly from one-another, and white boxes show the value of node strength. HRQL = health-related quality of life, GAD = anxiety, PHQ = depression, WS = work-related support, PS = personal support, WC = work-related concern, PC = personal concern.

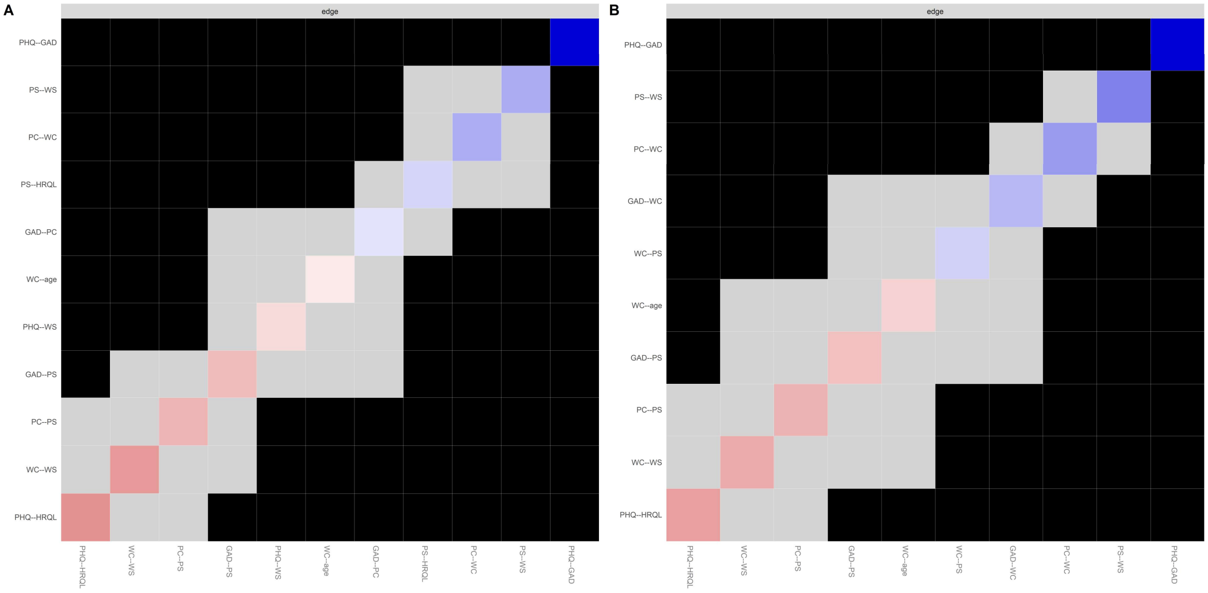

Figure S5. Edge weights difference test for the female (left) and male (right) network. Edge weights are shown in the diagonal. Black boxes represent significant differences between edge weights. HRQL = health-related quality of life, GAD = anxiety, PHQ = depression, WS = work-related support, PS = personal support, WC = work-related concern, PC = personal concern.
